# Trends in digital detection for the quality and safety of herbs using infrared and Raman spectroscopy

**DOI:** 10.3389/fpls.2023.1128300

**Published:** 2023-03-21

**Authors:** Rongqin Chen, Fei Liu, Chu Zhang, Wei Wang, Rui Yang, Yiying Zhao, Jiyu Peng, Wenwen Kong, Jing Huang

**Affiliations:** ^1^ College of Biosystems Engineering and Food Science, Zhejiang University, Hangzhou, China; ^2^ School of Information Engineering, Huzhou University, Huzhou, China; ^3^ College of Mechanical Engineering, Zhejiang University of Technology, Hangzhou, China; ^4^ College of Mathematics and Computer Science, Zhejiang A & F University, Hangzhou, China

**Keywords:** Infrared and Raman spectroscopy, rapid detection, natural products, herbal nutraceuticals, herbal medicine

## Abstract

Herbs have been used as natural remedies for disease treatment, prevention, and health care. Some herbs with functional properties are also used as food or food additives for culinary purposes. The quality and safety inspection of herbs are influenced by various factors, which need to be assessed in each operation across the whole process of herb production. Traditional analysis methods are time-consuming and laborious, without quick response, which limits industry development and digital detection. Considering the efficiency and accuracy, faster, cheaper, and more environment-friendly techniques are highly needed to complement or replace the conventional chemical analysis methods. Infrared (IR) and Raman spectroscopy techniques have been applied to the quality control and safety inspection of herbs during the last several decades. In this paper, we generalize the current application using IR and Raman spectroscopy techniques across the whole process, from raw materials to patent herbal products. The challenges and remarks were proposed in the end, which serve as references for improving herb detection based on IR and Raman spectroscopy techniques. Meanwhile, make a path to driving intelligence and automation of herb products factories.

## 1 Introduction

Herbs, referred to its raw materials, have been used as natural remedies for disease treatment, prevention, and health care after regulated processing, with a surge in acceptance and public interest rising. The treatment and prevention of herbs have been widely used worldwide since ancient times (Bonifacio et al., 2014). The significant achievement that artemisinin extracted from *Artemisia annua* for curing malaria was even awarded Nobel Prize in Physiology or Medicine (Tu, 2016). Herbal medicine, which is made from herbs, also plays an irreplaceable role in infectious diseases, which is confirmed in combating SARS (Xiao et al., 2003) and COVID-19 (Huang et al., 2020; Liu et al., 2020; Yang et al., 2020), by means of analysing and comparing clinical curative effects. The World Health Organization stated that about 80% of the world’s population relies on herbs for health care (Rohman et al., 2014). Some herbs contain active ingredients with functional properties that can be used as food or food additives, named medicine food homology plants (Granato et al., 2017). For example, *Curcuma longa* and *Lycium barbarum* are well-known traditional herbs serving tonic food due to their bioactive components (Xie et al., 2016; Tsuda, 2018). In addition, licensing systems have been established to ensure the marketing of qualified herbs (Ekor, 2014).

There will be health issues, safety risks, and abnormal market orders without requisite quality regulation. Therefore, the quality and safety inspection of herbs is essential, which is beneficial to guarantee the clinic’s effectiveness as well as decrease side effects. As herbs are natural plants, unlike synthetic drugs with clear ingredients, the quality and safety are influenced by various factors, such as habitat, maturity, and processing methods throughout the whole process of herbs, from raw materials to patent herbal products. Each unit is needed to be detected and controlled (Mackey and Nayyar, 2016; Li et al., 2021). The herb management of quality control mainly includes (1) identification of the authentication; (2) classification of the differences caused by geographical origin, species, and processes; (3) determination of the phytochemical constituents.

Traditional methods of quality control depend on a person’s knowledge or experience. The morphological and histological methods are vulnerable. Chromatography analytical methods, such as high-performance liquid chromatography (HPLC) and liquid or gas chromatography-mass spectrometry (LC/GC-MS), require skilled operation and complex processes, which is time-consuming without quick response and limits digital development in the modern herb industry. Therefore, rapid, non-destructive, and environment-friendly analytical strategies are current key points to make access to data acquisition and processing automatically, then boost intelligent and green development with immediate detection and instant decision required.

Infrared (IR) and Raman spectroscopy, the vibrational spectroscopy techniques can provide comprehensive chemical profiles of multiple compounds, characterizing the composition and content of target matter with objective, high-speed, and non-damage, which are regarded as effective tools in the field of herbs (Zou et al., 2005; Rohman et al., 2014; Chiachi, 2016; Kucharska-Ambroej and Karpinska, 2019). Besides, due to their advantages of non-damage detection, quick-response, and in-line analysis, IR and Raman spectroscopy techniques have broad application prospects in quality control and safety inspection of herbs, promoting the efficiency and accuracy of digital detection in the herb industry.

**Figure 1:**
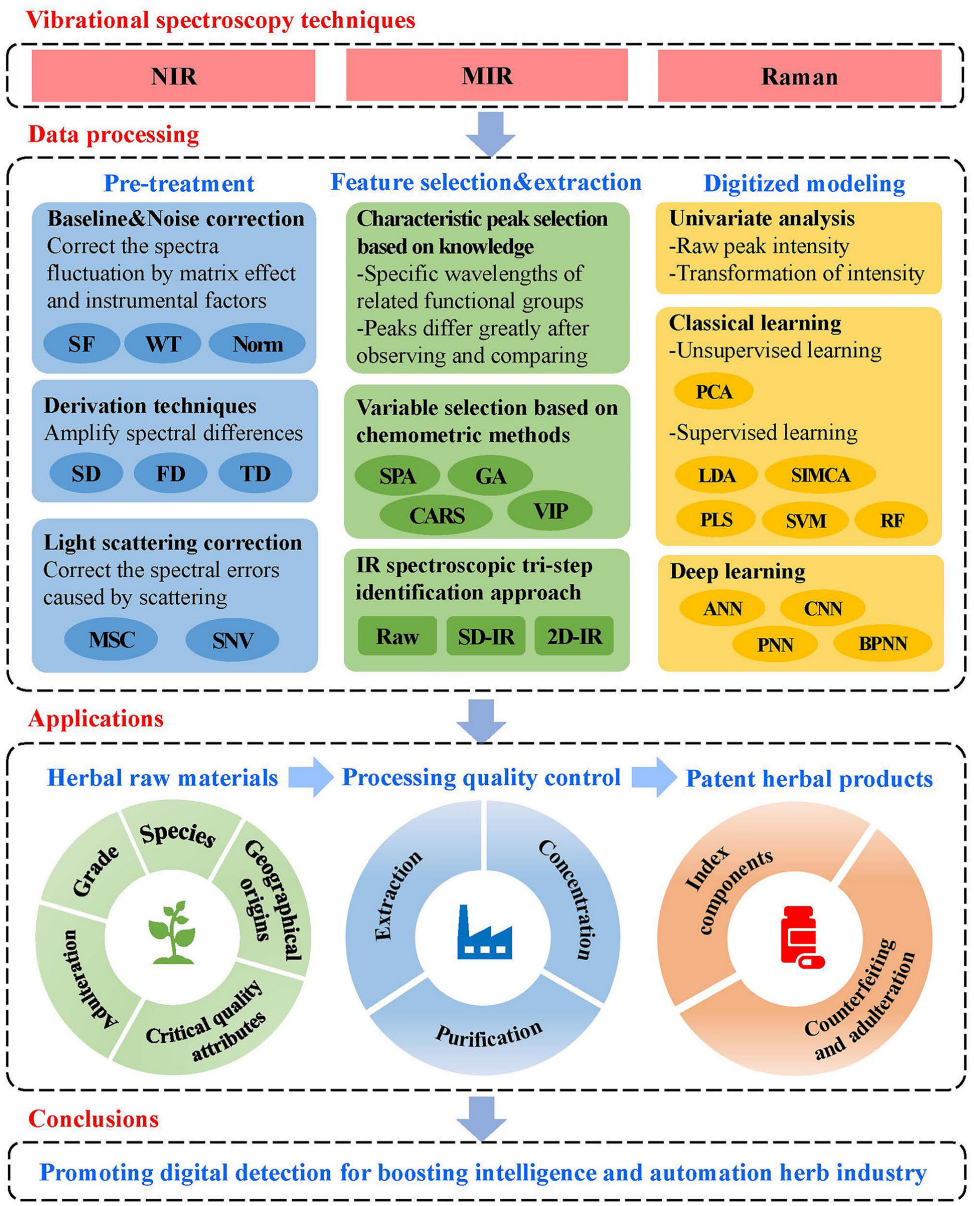
The framework of the review.

This review highlighted applying IR and Raman spectroscopy techniques in quality and safety inspection across the whole process of herbs, from raw materials to patent products. The framework of this review is shown in **Figure 1**. Firstly, a brief introduction to vibrational spectroscopy techniques and data processing methods was available. Secondly, the current application of herbs using IR and Raman spectroscopy techniques was presented from three aspects: (1) herbal raw materials; (2) processing quality control; and (3) patent herbal products, covering the whole process of herb production. Finally, we discussed the benefits and limitations of vibrational spectroscopy techniques. Several suggestions were put forward to improve the digital detection of the quality and safety of herbs.

## 2 Introduction of vibrational spectroscopy techniques

### 2.1 IR spectroscopy technique

IR spectroscopy studies the interaction between matter and infrared radiation. The main principle is that IR light’s energy could trigger the mechanical motion of specific molecular bonds when the IR light passes the sample, which is called IR absorption. A specific characteristic absorption presented in the IR spectra is employed for analysis according to the absorption frequency of chemical bonds and functional groups (Stark et al., 1986). The mechanical motion (vibration and rotation patterns) of atoms connected by covalent bonds includes symmetric and asymmetric stretching and scissoring, wagging, rocking, and twisting (Johnson and Naiker, 2020). IR spectroscopy contains richer group information with tremendous advantages in analysing and identifying organic substances, which has been widely used since the 1960s (Smith, 2011) and can be used for both qualitative and quantitative analysis (Stuart, 2005). The ingredients of concern in herbs, such as saponins, polysaccharides, flavonoids, triterpenoids, and polyphenols, consist of various organic functional groups that contribute to characteristic bands or peaks in IR spectra. The differences among spectra can be conducted to analyse the quality discrepancies of herbs. In the field of herbs, IR spectroscopy technique has been used since the early 1980s (Zou et al., 2005). Nowadays, IR spectroscopy is the most widely used technology in the quality detection of herbs, such as the identification of species, origins, grades, and the prediction of compound contents (Yin et al., 2019).

A typical IR spectrometer comprises a radiation source, a wavelength selection device, a sample holder, a photoelectric detector, and a computer system (Porep et al., 2015). The spectra acquisition modes include transmission, reflection, transflection, and interaction, which differ in how the detectors are placed with respect to the samples (Alander et al., 2013; Monnier, 2018). The IR region is conditionally divided into three subregions, including near-infrared (NIR, 12,820-4,000 cm^-1^), mid-infrared (MIR, 4,000-400 cm^-1^), and far-infrared (FIR, 400-33 cm^-1^). The quality analysis of herbs mainly focuses on NIR and MIR spectra caused by molecular vibration. FIR spectra excite molecular rotation and have strong water absorption, which is more suitable for heavy metal analysis (Su and Sun, 2018).

Various chemical bonds related to fundamental vibrations of molecules could be detected in MIR spectroscopy. The number of scans, resolution, and scan regions are vital parameters that affect signal-to-noise ratio (SNR) and spectra quality. The MIR region can be divided into two distinct regions, 4,000-1,500 cm^-1^ and 1,500-400 cm^-1^, which are called functional group region and fingerprint region, respectively. In the fingerprint region, 4,000-2,500 cm^-1^ is X-H (where X is C, N, O, or S) stretching vibration. 2,500-2,000 cm^-1^ corresponds to triple bonds, such as C≡C, C≡N). Double-bonded functional groups, like C=C, C=O, C=N, mainly lie in 2,000-1,500 cm^-1^. The peak near 3340, 1739, and 1670 cm^-1^ were assigned to the stretching vibration of O-H, ester carbonyl groups, and C=C, respectively, in the IR spectrum of *Dictamnus dasycarpus* Turcz (Liu et al., 2020). The peaks at 1684, 1517, and 1031 cm^-1^ were observed and compared to distinguish the different *Rhodiola* species (Tang et al., 2020). The region of 1200-950 cm^-1^ was chiefly assigned to the vibration of C-O related to polysaccharides (Wu et al., 2019).

MIR spectra are collected mainly by Fourier transform infrared (FTIR) spectrometers, which are equipped with a Michelson interferometer instead of the traditional grating monochromator, significantly improving the scanning speed, SNR, and the wavelength resolution of MIR spectroscopy. The advantages of FTIR are as follows: (i) non-destructive or only slightly damages the sample; (ii) needed sample quantities are small for measuring; (iii) requires minimal sample preparation at most. Meanwhile, the shortcomings of spectrum complication, quantification, and sample constraint are needed to be considered. Spectrum complications and quantification are solved by digitalisation and chemometric methods. More advanced FTIR techniques are developed to overcome the sample constraint, by which samples do not undergo time-consuming preparation that lets samples be combined with KBr. ATR is a sampling technique that is used to obtain high-quality data on liquid and solid. ATR-FTIR relies on the total internal reflection of infrared light in an internal reflection element or crystal with a high reflection index in direct contact with the measured sample, simplifying sample preparation (Feng et al., 2013). Amazing consistent sampling and higher accuracy may be achieved due to the presence of multi-reflective crystals, in which light is reflected on the sample many times, thereby increasing the absorbance (Lohumi et al., 2015). The limitation of ATR-FTIR is that it is challenging to achieve an ideal optical fit between the sample and the ATR crystal (Monnier, 2018). Diffuse reflectance FTIR (DRIFT) spectrometers and FTIR photoacoustic spectroscopy (FTIR-PAS) are developed for the direct determination of powder samples. The diffuse reflection accessories can collect the diffuse reflected light with absorption-attenuation characteristics caused by an uneven or rough surface, which obtains spectral signals with a good SNR to the maximum extent (Huang et al., 2008). DRIFT is suitable for the surface structure analysis of opaque or irregular solid samples. The advantage is that almost no preparation is required for the sample, which can be in powder form or film. FTIR-PAS collects spectral data from the pressure fluctuations generated by thermal expansion, which is detected by a sensitive microphone. Photoacoustic techniques mainly include modulated excitation and generation of sound waves in gaseous samples, modulated excitation of liquid and solid samples with an indirect generation of sound waves in the adjacent gas phase, and pulsed excitation and generation of pressure pulses in liquid and solid samples (Schmid, 2006). Rather than focusing on what is transmitted or reflected, FTIR-PAS measurement relies on the energy absorbed by samples, making it suitable for high-scattered, opaque, weak-absorbed, and low-concentration samples (Du and Zhou, 2011).

Unlike almost all modern MIR spectrometers based on Fourier transform, monochromator/detector principles in scanning NIR spectroscopy are variable. NIR spectroscopy lies between visible and infrared light, comprising broad bands associated with molecular overtones and combinations of vibrations. According to different combinations, simple molecules with few basic vibration modes can present many overtones in the NIR spectroscopy. NIR is sensitive to hydrogen groups such as O–H, N–H, and C–H (Wang et al., 2016). Therefore, the moisture of samples is needed to be considered (Buening-Pfaue, 2003). Infrared signals are easier to detect, but the overlapping of NIR spectra will affect the interpretation. As a result, NIR spectral data is analysed with a combination of chemometric methods to extract valuable information.

### 2.2 Raman spectroscopy technique

The change in the frequency of light scattered by molecules as it travels through a medium is called Raman scattering, discovered by C.V. Raman in 1928, relying on the inelastic scattering of photons known as Raman scattering (Raman and Krishnan, 1928). Raman scattering is a combined light scattering phenomenon produced by the interaction of light and matter molecules. The principle of Raman spectroscopy is analysing the scattering spectra with different frequencies from the incident light, which is applied to the study of the molecular structure of matter in specific wavenumber (Qin et al., 2019). Raman spectra cover a range of 4,000-50 cm^-1^. The advantages of Raman include sensitivity to chemical structure within the fingerprint regions and easy analysis without pre-treatment. Besides, due to the weak Raman scattering of water, Raman can be applied in an aqueous environment. The vibrations of various functional groups in herbs produce peaks at different positions due to unique spectroscopic fingerprints. The information of a class of chemical compounds with similar molecular structures can be deduced. For example, the peak at 1626 cm^-1^ is assigned to the stretching vibration of C=C bonds (Wong et al., 2015). Adulterations can also be recognized by comparing the peaks in Raman spectra. Therefore, the detection of species, adulteration, and ingredients, as well as processing monitoring using Raman spectra, is a feasible application in the field of herbs.

A typical Raman spectrometer consists of five components: laser light source, filter, sample cell, monochromator, and detector (Eliasson et al., 2008). There are many types of lasers, ultraviolet laser, visible laser, and NIR laser, available to be applied. The selection of laser depends on samples and detection purposes, which can be considered in three aspects. (i) The intensity of the Raman signal. According to the acknowledged relationship, I_Raman_∝1/λ^4^, the shorter wavelength of the laser, the stronger the Raman signal. (ii) Avoid fluorescent interference to prevent the annihilation of the Raman signal by fluorescent signal. Choosing an excitation laser outside the fluorescent region, like an ultraviolet laser or NIR laser, can avoid the fluorescence effect. (iii) The need to analyse samples at different depths. The longer the wavelength of the laser, the deeper the penetration (Lee et al., 2013). Basic Raman measurement techniques contain backscattering, transmission, and spatially offset Raman spectroscopy (SORS) (Qin et al., 2019). The backscattering collection mode is mainly used for sample surface inspection. The transmission collection mode is more suitable for the bulk composition of samples that are non- or weak-absorbing inside (Eliasson et al., 2008). SORS has the capability to obtain layered information on samples by setting a series of lateral offsets (Nicolson et al., 2021). Generally, the selection of lasers and measurement modes is based on the characteristics of the samples. Shorter excitation wavelengths could excite stronger Raman signals, but higher energy damages the sample more. Meantime, the cost and volume of the instrument increase.

The main disadvantages of Raman spectroscopy are the thermal effects of the sample, fluorescence interference, and weak Raman signals (Wang et al., 2018). Such obstacles could be overcome by the advancements in devices and materials (Chen et al., 2017), which lead to a greater variety of analytical techniques (Gala and Chauhan, 2015). Fourier transform Raman spectroscopy (Liao et al., 2004), resonance Raman spectroscopy (RRS) (Robert, 2009), confocal Raman spectroscopy (Barbillat et al., 1994), and surface-enhanced Raman spectroscopy (SERS) (Sharma et al., 2012; Pérezjiménez et al., 2020), is feasible to enhance Raman signals by 10^3^-10^6^ times, which evolves the instruments and samples processing. FT-Raman adopts Fourier transform technique and is equipped with a NIR laser (1064 nm) as an excitation light source that avoids fluorescence interference. But its baseline drift and poor reproducibility affect its Raman signal. RRS depends on the resonance effect that the frequency of the laser matches an electronic transition of the irradiated molecule. Its instrument requires an adjusted light source. Confocal Raman spectroscopy which is the coupling of Raman to microspectroscopic instruments, can provide a high-resolution image rich in information. The development of techniques and instruments creates more practical applications in Raman spectroscopy.

In the field of herbs, SERS has become a hotspot for analysis. Fleischmann et al. (1974) discovered SERS during measurements of Raman scattering of pyridine on rough silver electrodes. SERS amplifies conventional Raman signals by combining nanostructures of noble metals with the sample, whose theoretical mechanisms involve electromagnetic enhancement and chemical enhancement. Nanomaterials improvements and chemical modifications offer more possibilities for SERS applications, advancing towards selectivity, *in situ*, and non-destructive sampling detection (Lin and He, 2019; Langer et al., 2020), which has achieved feasible applications in adulteration detection (Dao et al., 2019), compound identification (Gu et al., 2018), and on-site qualitative screening (Zhu et al., 2014). The complex matrix effect and limited multi-analyte capability are needed to be considered. Nevertheless, the great compatibility with other techniques, such as separation techniques and other innovations and variants of Raman spectroscopy, makes SERS promising.

### 2.3 Comprehensive comparison of IR and Raman spectroscopy in herbs

**Figure 2:**
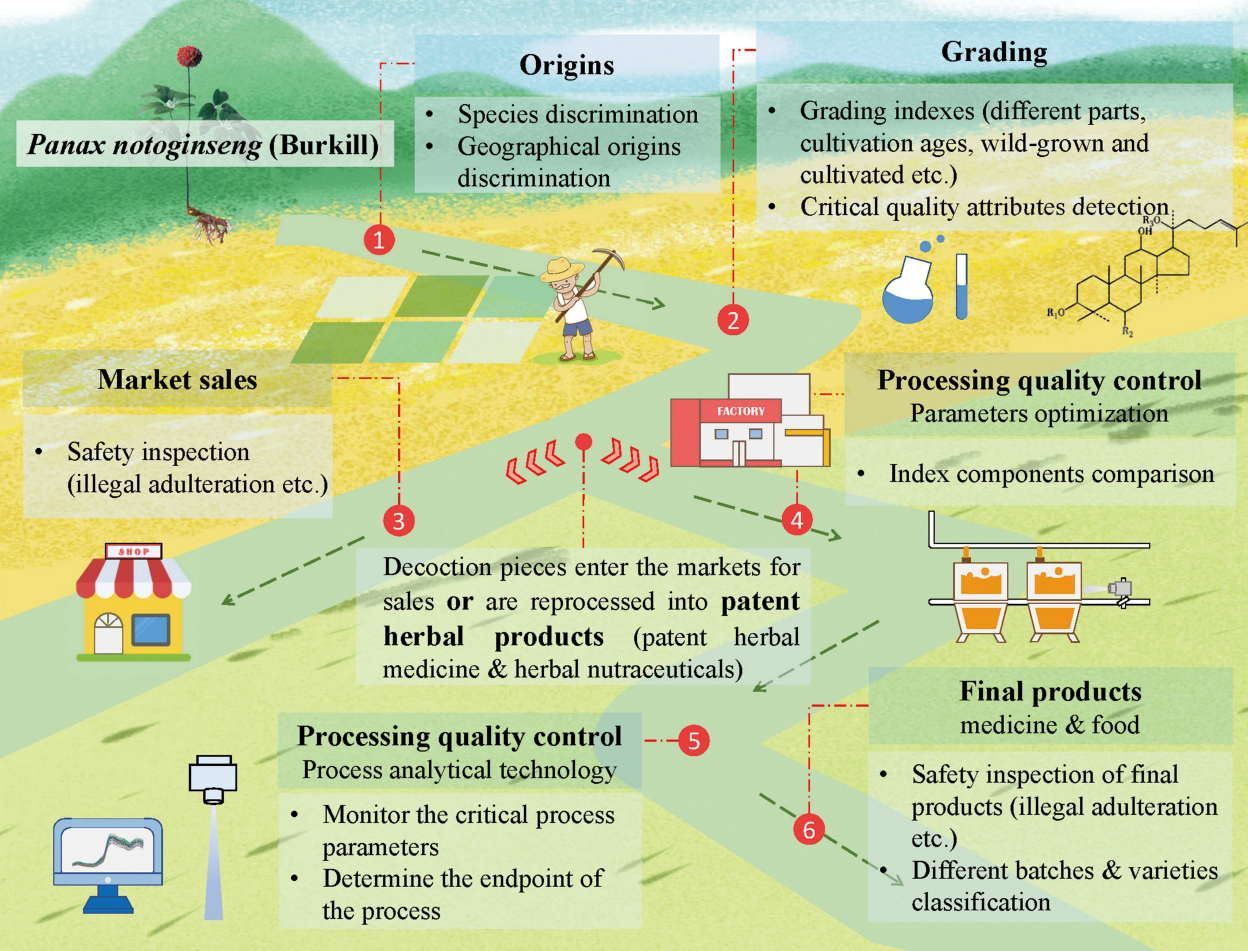
The quality and safety inspection of herbs across the whole process (set *Panax notoginseng* as a typical case).

In the field of herbs, clinical efficacy is due to multiple compositions working in concert. IR and Raman spectra that reflect the comprehensive chemical profiles related to composition are feasible to be applied in the qualitative analysis of herbs, including identifying the species, grades, origins, and quantitative prediction. In **Figure 2**, *Panax notoginseng* is selected as a typical case, and IR and Raman spectroscopy techniques are adopted for quality and safety inspection throughout the process. The comparison of IR and Raman spectroscopy is summarized in **Table 1**. Each technique has its own advantages and disadvantages. The detection method selection should be based on sample characteristics and detection purpose. Herb materials’ compounds are complex, and IR and Raman spectroscopy techniques complement each other. IR spectroscopy detects the molecule with IR absorption when its dipole moment changes. The molecular bond without dipole moment but with polarizability change can be detected in Raman spectroscopy. The characteristic peak information of MIR spectroscopy is pointed to specific databases that are relatively complete. Meanwhile, as mentioned in section 2.2, the poor spectral reproducibility and SNR in Raman spectra were caused by the fluorescence and sample matrix effect, which leads to the status that Raman spectroscopy is less widely used than IR spectroscopy in herbs (Woo et al., 1999c).

**Table 1:** Comparison of IR and Raman spectroscopy.

Techniques	NIR	MIR	Raman
**Wavenumber**	12,820-4,000 cm^-1^	4,000-400 cm^-1^	4,000-50 cm^-1^
**Principle**	Infrared absorption	Infrared absorption	Light scattering
**Produce conditions**	Molecular dipole moment changes	Molecular dipole moment changes	Molecular polarizability changes
**Spectra shape**	Broad bands	Sharp absorption peaks	Sharp spectral peaks
**Sample types**	solid, liquid, gas		
**Sample preparation**	Non or minimal		
**Applications**	qualitative and quantitative analysis		
**Light source**	(Dispersed) Polychromatic radiation; globar tungsten		Monochromatic radiation; laser
**Group preference**	Polar bond vibrations of different atoms		Non-polar bond vibrations of the same atom
**Container**	Cannot be measured in glass containers		Can directly be measured in glass bottles and capillaries
**Structure analysis**	NO	YES	YES
**Moisture interference**	YES	YES	NO
**Limitation**	Bands overlapping	Sample constraint	Fluorescence interference; thermal effect

IR and Raman spectroscopy techniques are available to detect samples that are in the original state without sample pre-processing. However, simple sample preparation is employed before collecting spectral data to gain high-quality spectra and better analysis results. Dried samples after grinding and tableting, or extracts, are two commonly used herbal raw materials for analysis, which decreases the matrix effect. Digital technology offers a data processing method to remove irrelated variables, which makes sample pre-processing unnecessary. SPA-LDA algorithm extracted seven effective variables to achieve the three origins discrimination of *Ginseng* in piece form using NIRS (Chen et al., 2020b).

Spectral analysis can realize the quality identification and evaluation of herbs with different qualities. Currently, the accuracy and precision of herb detection using IR and Raman spectroscopy techniques are not up to the standard analysis methods. The outstanding advantages of rapid, accurate, and online analysis endow the application prospect of the digital detection and automation industry.

## 3 Introduction of data processing in digital detection

**Figure 3:**
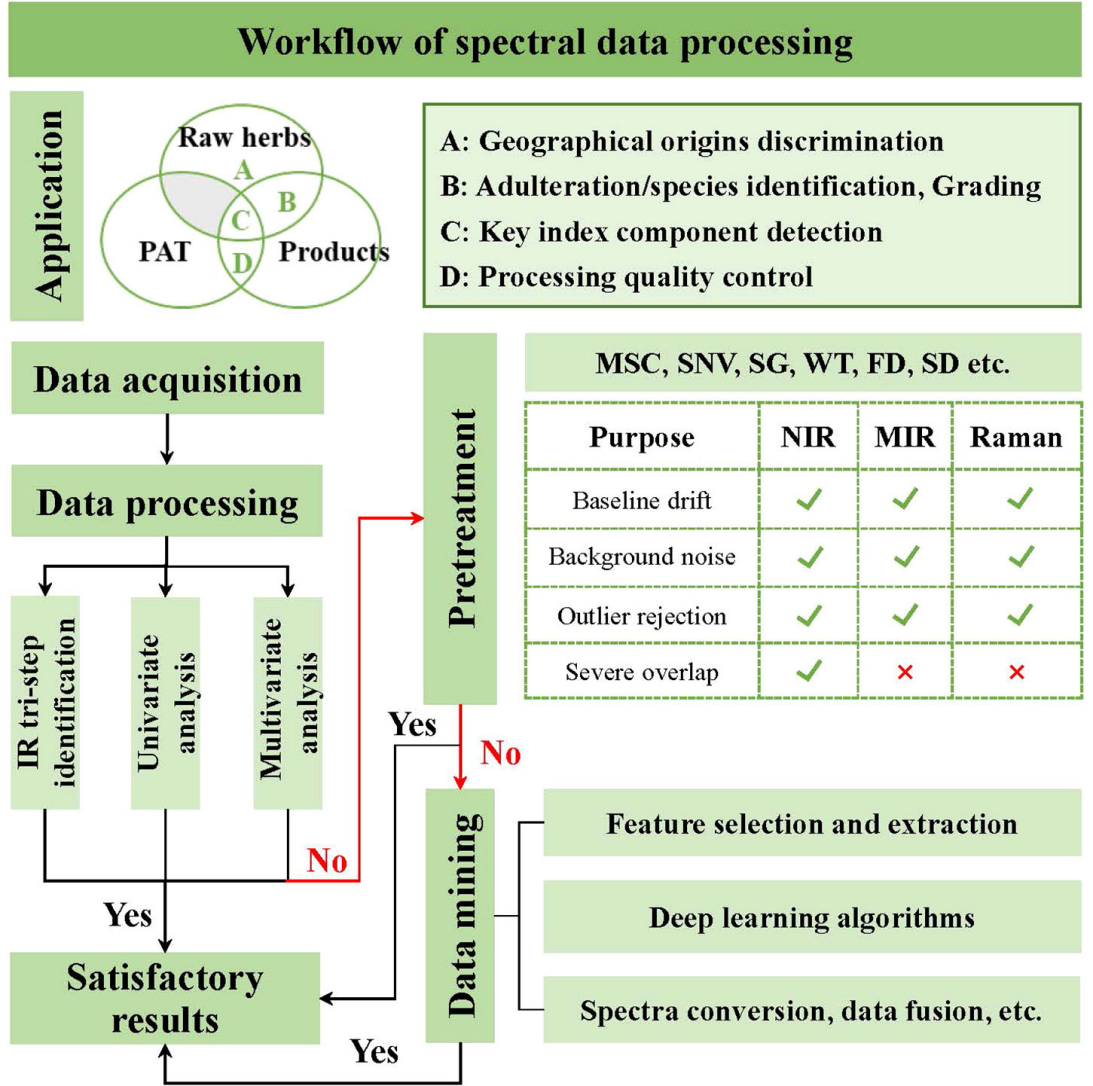
The rough schematic diagram of spectral data processing.

Vibrational spectroscopy techniques are easily accessible to acquire data. The robust models are established in the way that spectral data as input and class labels or predicted value as output, which aims at achieving digital detection with the real-time response by digital technology (Li et al., 2021). **Figure 3** presents the workflow of spectral data processing. For the purpose of improving accuracy and sensitivity, the attempts, data pre-treatment, and feature selection usually are carried out.

### 3.1 Pre-treatment

Influenced by samples’ physical factors (compactness, smoothness, particle size, etc.), instrument error, and the experimental environment, IR and Raman spectroscopy inevitably present some irrelevant information to the target samples, which results in baseline drift, spectral overlap, and background noise. Therefore, spectral pre-treatment is used to remove defects observed in the spectra and amplify the differences in the raw spectra of samples (Baker et al., 2014).

After many attempts, baseline and noise correction (Liu and Yu, 2016), derivation techniques (Rinnan, 2014), and light scattering correction (Buddenbaum and Steffens, 2014) are considered effective pre-treatment methods, which are common strategies in the spectral detection of herbs. Baseline correction (BC) is widely applied to correct the spectra by particle size variation and instrumental factors (Cadet and Offmann, 1996). It needs to be noted that variable baseline signals and cosmic rays in Raman spectra contribute to spectral contamination causing less sensitivity. Baseline correction and cosmic ray removal are the primary purposes of Raman spectra pre-treatment (Li et al., 2015). Noise correction helps to improve the signal-to-noise ratio (Liu et al., 2019). Smoothing and filtering (SF), wavelet transform (WT), and normalization (Norm) are applied to correct the baseline or reduce noise. Common derivative pre-treatments include the first derivative (FD), second derivative (SD), and third derivative (TD), which are used to amplify spectral differences. Multiplicative scatter correction (MSC) and standard normalized variate (SNV) are applied to correct the spectral errors caused by scattering.

Additionally, multiple pre-processing methods were combined to attain the best performance in studies (Rinnan et al., 2009). The effective and efficient improvements in models were compared to obtain the most optimal one for further analysis (Xu et al., 2019).

### 3.2 Feature selection and extraction

The data collected by vibrational spectroscopy techniques is generally too much and may result in redundant information interfering with the correlation, which is unfavourable to the establishment of the model. Extracting feature wavelengths is a practical approach to improve the robustness of the model. Though not an indispensable step when processing the data, variable selection is usually employed to remove or eliminate useless variables with noise and irrelevance, even interference. By using these methods, the bias caused by chemical, physical, and instruments decreases, which results in better predictions and simpler models (Zou et al., 2010). Simple identification tasks can even be realized directly by comparing the change rules of characteristic peaks.

#### 3.2.1 Characteristic peak selection based on knowledge

Regarding MIR and Raman, bands’ number, position, shape, and intensity vary with compounds and their aggregation states, and these characteristic peaks were adopted for determination, which makes for structure analysis and detailed interpretation (Wang et al., 2018). In the early days of IR spectroscopy technique for herbs, characteristic peak analysis was the typical method, which could be used for small-category identification. The distinct groups or chemical substances to be measured appear in specific wavelengths of related functional groups (Magwaza et al., 2012; Hadjiivanov et al., 2021), or the position where the peaks differ greatly after observing and comparing among various qualities of herbs. The fingerprint peaks can be as feature lines used for qualitative identification and quantitative analysis of substances directly.

#### 3.2.2 Variable selection based on chemometric methods

Feature selection and extraction help establish more reliable and practical models. Fewer variables also reduce the computer calculation time. Wavelength point selection methods and wavelength interval selection methods are effective in retrieving and selecting features in the spectra (Yun et al., 2019). Competitive adaptive reweighted sampling (CARS), successive projections algorithm (SPA), genetic algorithm (GA), variable influence on projection (VIP), and interval PLS (iPLS) are employed as different categories of variable selection methods (Song et al., 2017; Junaedi et al., 2021). The selected spectral variables that contributed to detection were related to crucial index compounds, providing scientific support to the relationship between chemical components and medicinal efficacy. It can be seen from the existing review literature that the complexity of variable selection methods is gradually increased since the evaluation indexes are more diverse. The improved variable selection strategy is a valuable guideline for establishing a real-time platform since we can set dozens of spectra as targeted regions. Scholars prefer to combine multiple methods for better performance (Pasquini, 2018).

#### 3.2.3 IR spectroscopic tri-step identification approach

The IR spectroscopic tri-step identification approach is a new model-free method (Noda, 1993) that focuses on feature bands for analysis. It consists of three steps: (1) raw infrared spectra; (2) the second derivative infrared (SD-IR) spectra; and (3) two-dimensional correlation infrared (2D-IR) spectra. The IR spectroscopic tri-step identification approach is utilized to resolve the overlapped signals and amplify spectral differences to obtain higher-resolution spectra.

SD-IR spectra can improve the apparent resolution, reduce the overlap of absorption peaks, and enhance the spectral characteristics of the low-energy components. The spectral fluctuations of 2D-IR spectra can be treated as an arbitrary function of almost any physical variable, such as temperature, time, concentration, and pressure, employed to expand to IR spectroscopy in a two-dimension, whereas these spectral features cannot be observed in conventional one-dimensional spectra. 2D-IR spectra include the synchronous spectrum (Guo et al., 2016) and the asynchronous spectrum (Miao et al., 2017).

The IR spectroscopic tri-step identification approach has gradually developed as a systematic analysis method in the herb detection of different forms, whose applications in identification and optimization with comprehensive and objective assessment are summarized in **Table 2**. This method provides a simple economic insight, which can be used as anterior guidance in detecting multi-level (from entirety to fraction to single ingredient) and multi-plex (from major to minor to trace components), especially in rare or expensive samples whore the number is too limited to build a pattern recognition model. Besides, the tri-step identification gives a holistic view of the herbs, which supplements conventional methods that focus on several selected marker substances and neglect the synergistic effect. An analysis-through-separation approach was proposed to provide a pyramid of chemical fingerprints in *Salviae miltiorrhizae* (Xu et al., 2013).

**Table 2:** Summary of feature analysis of herbs based on IR spectroscopic tri-step identification approach.

Herbal plants	Technique	Application	Data processing	Ref.
*Astragalus membranaceus*	FTIR	Geographical origins discrimination	SD/2D-IR	Huang et al. (2015)
*Carthamus tinctorius*	FT-IR/NIR	Adulteration identification	BC+PCA/SD/2D-IR	Chen et al. (2015)
*Cordyceps sinensis*	FTIR	Geographical origins discrimination	SD/TOPSIS	Sun et al. (2019)
*Dendrobium officinale*	FTIR	Desiccation methods optimization	SD/2D-IR	Wu et al. (2019)
*Dictamnus dasycarpus*	FTIR	Cultivation ages discrimination	SD	Liu et al. (2020)
*Eurycoma longifolia*	FTIR	Extraction process optimization	SD/2D-IR	Adib and Abdullah (2018)
*Fritillaria thunbergii*	FTIR	Adulteration identification	SNV+2D-IR+SIMCA	Chen et al. (2018)
*Ganoderma*	FTIR	Index compounds analysis	SD/2D-IR	Choong et al. (2011)
*Gardenia jasminoides*	FTIR	Thermal process optimization	SD-IR/2D-IR/PCA	Chen et al. (2016)
*Ligusticum*	FTIR	Processing discrimination	Smoothing+SD+2D-IR	Guo et al. (2016)
*Lonicera japonica*	FTIR	Species discrimination	SD/2D-IR	Yan et al. (2016)
*Lycium barbarum*	FT-NIR	Geographical origins discrimination	2D-IR	Lu et al. (2008)
*Panax ginseng*	FTIR	Cultivation ages discrimination	SD/WT+2D-IR	Zhan et al. (2007)
	FTIR	Species discrimination	SD-IR+2D-IR	Lu et al. (2008)
	FTIR	Cultivation types and ages discrimination	BC/SD+SIMCA/2D-IR	Zhang et al. (2010)
*Prunus*	FTIR	Processing discrimination	2D-IR/HCA	Cheng et al. (2017)
*Rheum*	ATR-FTIR	Stir-baking process optimization	SNV+2D-IR/PCA	Yang et al. (2020)
*Rhodiola crenulata*	FTIR	Species discrimination	BC/Norm+SD/2D-IR/PCA/PLS-DA	Tang et al. (2020)
*Salvia miltiorrhiza*	FTIR	Chemical characterization demonstration	SD	Xu et al. (2013)
*Scutellaria baicalensis*	FTIR	Optimal harvesting season determination	SD	Xu et al. (2013)
Patent herbal medicines	FTIR	Adulteration identification	SD/2D-IR	Miao et al. (2017)
Patent herbal preparations	FTIR	Quality control standard analysis	SD/2D-IR	Chen et al. (2007)

### 3.3 Digitized modeling

Chemometrics combined with vibrational spectroscopy techniques is an effective tool that can be understood as applying mathematics and statistics to chemical data processing (Rohman, 2019). Conventional statistics focus on a particular situation based on predefined distributions and model assumptions, not properly applied to every kind of data (Breiman, 2001). Digitized modeling can be improved and perfected continuously to expand the scope of application, building more robust models with higher accuracy.

#### 3.3.1 Univariate analysis

Univariate analysis is a conventional method that is built from characteristic peaks. Peaks are observed to analyse differences or establish calibration curves, which achieve qualitative or quantitative goals. The selection of one or more fingerprint characteristic peaks or the transformation of band intensity (intensity ratios, etc.) from MIR or Raman spectroscopy will help form a spectral input matrix for comparison and analysis (Li and Church, 2014; Peng et al., 2015; Byrne et al., 2016).

#### 3.3.2 Multivariate analysis

Multivariate analysis with more information, which helps to dig out the relation between spectra and substance, has achieved acceptable results in classification and detection (Jan et al., 2015; Nturambirwe and Opara, 2020). There are two main categories of machine learning (ML), unsupervised and supervised learning (Kavakiotis et al., 2017). In the quality and safety inspection of herbs using vibrational spectroscopy techniques, principal component analysis (PCA), linear discriminant analysis (LDA), soft independent modeling of class analogy (SIMCA), partial least squares (PLS), support vector machine (SVM), and random forest (RF) are often used (Jimenez-Carvelo et al., 2019; Kucharska-Ambroej and Karpinska, 2019; Zhang et al., 2020). Validation and prediction are required to verify models’ performance and general applicability (Ralbovsky and Lednev, 2020).

Undoubtedly, distinguishing and extracting useful information from a large amount of spectral data is the key to building an ideal model. With the development of computational systems, a new paradigm named deep learning (DL) provides more general models for detection than shallow approaches. DL network performance is better in feature mining, which is more suitable for complicated data with unclear features. DL is a representation-learning method that autonomously learns relevant and deep features of input information, showing a great preponderance of extracting the features among complex data (Zhou et al., 2019; Lussier et al., 2020). Artificial neural network (ANN) (Liu et al., 2019), convolutional neural network (CNN) (Dong et al., 2019), back propagation neural network (BPNN) (Yang et al., 2018), and probabilistic neural network (PNN) (Du et al., 2017) have been applied in the field of herbs with satisfactory performance.

In addition, new ideas, like the conversion of spectra and data fusion, are proposed as processing strategies for data mining, which contributes to improving the results. Spectroscopy-image conversion is a popular idea to promote smart identification. An innovative chemometric modeling-free near-infrared barcode strategy was proposed by comparing the percentage of nonzero overlap between standard samples’ barcodes and samples needed to test (Dong et al., 2020). The spectral matrix was transformed into an image, and data augmentation techniques expanded the sample scale (Dong et al., 2019). The similarities in images lead to misjudgements. More representative samples are needed to acquire more feature information in further research.

Data fusion is divided into low-, mid-, and high-level (Wu et al., 2018). The low-level fusion contains a lot of useless or even interfering information, which hinders the synergistic effect of the multi-sensor information fusion strategy. Usually, we at least employed the mid-level fusion method to obtain satisfactory results. Data from different spectrometers, such as NIR and MIR (Fu et al., 2019; Pei et al., 2019; Zhou et al., 2020), FTIR and UV-vis (Wang et al., 2020), MIR and Raman (Wong et al., 2015; Wang et al., 2020), or various experiment materials collected from the same plants, such as with and without tunic (Biancolillo et al., 2020) and different botanical parts with its classifiers processing model (Liu et al., 2020) were merged to prove the feasibility of data fusion. Meanwhile, Both IR and Raman spectroscopy can be combined with imaging technology to obtain pixel-level image features. Data analysis can be done by combining spectra and image information to characterize samples more comprehensively (Flach and Moore, 2013; Araujo et al., 2018).

## 4 Quality and safety inspection of herbal raw materials

### 4.1 Species discrimination

Authenticity is the primary importance, which is the first step in the whole process of herb production. Herbs that belong to the same genus, even the same family, have a similar appearance. However, the value of herbs from various species is definitely different. The species’ characteristics make it impossible to be used interchangeably. The difficulty of species discrimination causes the phenomenon of counterfeit products and the misuse of raw materials. IR and Raman techniques, the feature bands presented in the spectra can be analysed to identify species.

Species discrimination using NIR reflectance spectroscopy was dated back to 1999 in *Ginseng radix et rhizome* (Woo et al., 1999b). The identification of *F. thunbergia* Miq from the genus *Fritillaria* (Meng et al., 2015), peach and apricot kernels (Kajino et al., 2021), the extracts of *Ganoderma lucidum* and *Vesicolor* (Shao et al., 2015), and *Eleutherococcus senticosus* from other eight herbs (Lucio-Gutiérrez et al., 2011) using NIR spectra was achieved. High discrimination accuracy in *Ginseng* (Yap et al., 2007) and Lingzhi species (Wang et al., 2019) was obtained based on FTIR combined with ML. SVM correctly discriminated against two species by the MIR and NIR model (Chen et al., 2020a). Gao et al. (2005) illustrated the feasibility of identifying different species of *Fritillariae bulbus* by convolution transform visualization fingerprint. Detection techniques, applications, and specific data processing methods of different herb species are concluded and listed in **Table 3**.

**Table 3:** Summary of qualitative analyses of herbal raw materials based on IR and Raman spectroscopy.

Herbal plants	Technique	Application	Data processing	Ref.
*Ganoderma*	DR-FT-NIR	Geographical origins discrimination	RF	Lai et al. (2018)
	NIR	Geographical origins discrimination	SD+PCA/MD/PLS-DA	Woo et al. (1999b)
	DR-NIR	Geographical origins discrimination	MSC/SNV/FD/SD+PCA/PLS-DA/MD	Chen et al. (2008)
	FTIR	Wild-grown and cultivated discrimination	SNV+PLDA/Elnet/PCA/PLS-DA	Zhu and Tan (2016)
	DR-FT-NIR	Species discrimination	PCA/PLS-DA	Shao et al. (2015)
	ATR-FTIR	Species discrimination	SD+RF/SVM/PLS-DA	Wang et al. (2019)
*Astragalus membranaceus*	FTIR	Adulteration identification	MC/Norm/MSC/FD+PCA/LDA/KNN/PLS-DA	Yang et al. (2020)
	FTIR	Adulteration/Geographical origins discrimination	BC/Norm+MDPLS-DA	Zhang and Nie (2010)
	SERS	Authentic and counterfeit medicine identification	PCA-LDA	Lin et al. (2018)
	FTIR	Geographical origins discrimination	SF/BC/DN/MSC+OPLS-DA/BP-ANN	Li et al. (2016)
*Panax ginseng*	NIR	Geographical origins discrimination	SNV/FD+PLS-DA/SIMCA/SPA-LDA	Chen et al. (2020b)
	FT-NIR	Geographical origins discrimination	MSC+SD+NIR barcode method	Dong et al. (2020)
	NIR/ FT-Raman	Geographical origins discrimination	SD+PCA/PLS-DA	Woo et al. (1999c)
	DR-NIR/ ATR-FTIR	Parts discrimination	FD+PCA	Wu et al. (2011)
	FTIR	Cultivation ages and parts discrimination	Norm+VIP+PLS-DA	Lee et al. (2017)
	FTIR	Cultivation ages and species discrimination	BC/Norm+PLS-DA	Kwon et al. (2014)
	NIR	Species discrimination	SD+WD/RV/MD/SIMCA	Woo et al. (1999a)
	FTIR	Species discrimination	BC/SD+PCA	Yap et al. (2007)
*Panax notoginseng*	NIR	Adulteration identification	SNV+Relief-based feature selection+ data-driven SIMCA	Chen et al. (2020a)
	NIR	Adulteration identification	SNV/FD/SD+PCA	Chen et al. (2019)
	NIR	Adulteration identification	Smoothing/SNV/MSC/FD/SD/CWT+HCA/PCA/PLS-DA/ANN/SVM/ELM	Liu et al. (2019)
	NIR	Geographical origins discrimination	Norm+SD+ CNN	Dong et al. (2019)
	FT-IR/NIR	Geographical origins discrimination	SD/SNV/SG+data fusion+RF	Zhou et al. (2020)
	NIR	Geographical origins discrimination	SNV/MSC/FD/SD+PLS-DA/SIMCA	Hui et al. (2018)
	FTIR	Geographical origins discrimination	BC/SF/Norm+SDA	Liu et al. (2015)
*Salvia miltiorrhiza*	FT-NIR	Geographical origins discrimination	SNV/MSC/SG/FD+PLS-DA	Sun et al. (2020)
	NIR	Geographical origins discrimination	MSC/SNV/FD/SD/ND/SG+PLS-DA	Wang et al. (2020)
	NIR	Geographical origins discrimination	SG/SD+local variable selection+PCA/SIMCA/PLS-DA	Zhu et al. (2018)
	NIR	Geographical origins discrimination	WD-IMA/KNN/LDA/QDA	Li and Qu (2014)
*Lonicera japonica*	FT-IR	Species discrimination	BC/SD/SG+PCA/LDA	Chen et al. (2017)
	NIR	Species discrimination	SNV/SD/ND+PCA/SIMCA	Li et al. (2013)
*Fritillaria thunbergia*	NIR	Species discrimination	Factorization method	Meng et al. (2015)
	DR-NIR	Species discrimination	Norm+CA+ convolution transform visualization similarity	Gao et al. (2005)
*Gastrodia elata*	NIR	Wild-grown and cultivated discrimination	SNV+Relief +PCA/PLS-DA/ELM/Adaboost.M1	Chen et al. (2021)
	FT-NIR	Adulteration and geographical origins identification	SD/SNV+OCPLS/PLS-DA	Li et al. (2017)
*Gentiana*	FTIR	Geographical origins discrimination	MSC/SNV/FD/SD/ND/SG+data fusion+PCA/PLS-DA	Liu et al. (2020)
	FTIR	Geographical origins discrimination	BC/SD/SG/Norm+SVM/PLS-DA	Wang et al. (2018)
*Poria cocos*	NIR	Geographical origins discrimination	SDD+PCA+CARS/MC-UVE/SPA/LPG+ PLS-DA/FDA	Yuan et al. (2018)
*Paris polyphylla*	FTIR	Species and geographical origins discrimination	MSC/SNV/SG/WT+VIP+PCA/PLS-DA	Yang et al. (2019)
	FT-IR/NIR	Geographical origins discrimination	SNV/FD/SD+PCA/RFE/Bo+PLS-DA/RF+data fusion	Pei et al. (2019)
*Chaenomeles speciosa*	NIR	Geographical origins discrimination	FD/SD/SNV/MSC+PLS-DA/HCA	Han et al. (2016)
*Acanthopanax senticosus*	NIR	Species and adulteration discrimination	SNV/SD+PCA/SIMCA/PLS-DA	Lucio-Gutiérrez et al. (2011)
*Angelica sinensis*	NIR	Geographical origins discrimination	SD+SIMCA	Woo et al. (2005)
	FT-NIR/MIR	Geographical origins discrimination	PCA/LDA/PLS-DA/MWPLS-DA	Fu et al. (2019)
*Corydalis*	FT-NIR/MIR	Adulteration identification	PCA/LDA/PLS-DA/MWPLS-DA+data fusion	
*Curcuma*	FTIR	Comprehensive quality control	SNV+PCA/HCA	Gad and Bouzabata (2017)
*Notopterygium incisum*	NIR/MIR/ E-nose	Species discrimination	Norm+PCA/SVM	Chen et al. (2020a)
*Allium sativum*	ATR-FTIR	Geographical origins discrimination	PLS-DA+data fusion (SO-PLS-LDA/SO-CovSel-LDA)	Biancolillo et al. (2020)
*Epimedium*	NIR	Geographical origins discrimination	FD/SG+DA/BPNN/KNN/SVM	Yang et al. (2018)
*Prunus*	NIR	Species discrimination	SD/SG/SNV+PCA/PLS-DA	Kajino et al. (2021)
*Eucommia ulmoides*	ATR-FTIR/ UV-vis	Geographical origins discrimination	FD/SD/TD/MSC/SNV/SG+PLS-DA/ GA-SVM/HCA+data fusion	Wang et al. (2020)
	NIR	Species discrimination	SNV+PCA/FDA	Lin et al. (2020)
*Lilium*	NIR	Species discrimination	MSC/SNV/SG/FD/SD+RF	Huang et al. (2020)
*Cordyceps sinensis*	FTIR-PAS	Geographical origins discrimination	SG+PCA+PNN	Du et al. (2017)
*Ganoderma*, *Lycium barbarum*, *Lonicera japonica*, and *Zanthoxylum*	SERS	Dye adulteration identification	Peaks analysis	Dan et al. (2015)
*Daemonorops draco*	SERS	Dye adulteration identification	Peaks analysis	Wu et al. (2018)
*Lonicera japonica*, *Chrysanthemum*, *and Rosa rugosa*	SERS	Dye adulteration identification	Peaks analysis	Liu et al. (2018)

The preliminary conclusion is drawn that the classification accuracy decreases with increasing categories, and the close geographical relationship makes it more challenging to discriminate. There are two main concerns to expanding the acceptance of IR and Raman spectroscopy techniques. One is model transferring capability (Li et al., 2013), and another is the miniaturization of devices (Lin et al., 2020). Chen et al. (2017) compared the benchtop and hand-held FT-IR equipment, pointing out that hand-held spectrometers had a promising prospect.

### 4.2 Geographical origins discrimination

Geographical origins are the second factor needed to be considered after species. Herbs are directly influenced by growing conditions like climate, soil, and altitude. The quality disparity of herbal raw materials from different origins exists. Where herbs are widely recognized with the highest value is called a geo-authentic area, and consumers appreciate herbs from the geo-authentic area. In this sense, geo-herbalism becomes the comprehensive evaluation criterion of excellent-quality herbs. Identifying the original source is beneficial to ensure quality consistency and avoid counterfeiting. IR and Raman spectroscopy techniques present the complete chemical profiles of herbs, paying more attention to the overall internal components.

Woo et al. (1999b) determined the geographical origins of *Astragali radix*, *Ganoderma*, and *Smilacis rhizome* with NIR reflectance spectroscopy in 1999. In the same year, Woo et al., (1999c) classified the cultivation areas of *Ginseng radix et rhizoma* using NIR and Raman techniques. From then on, scholars tried to achieve origins discrimination based on IR and Raman. Studies about the origin discrimination of herbs, like *Salviae miltiorrhizae radix et rhizoma*, *Paridis rhizoma*, and *Notoginseng*, have been carried out combined with various pattern recognition methods, including classic algorithms and innovative algorithms (Woo et al., 2005; Li and Qu, 2014; Liu et al., 2015; Han et al., 2016; Hui et al., 2018; Lai et al., 2018; Yang et al., 2018; Zhu et al., 2018; Fu et al., 2019; Yang et al., 2019). Detailed information containing techniques and data processing is listed in **Table 3**, and the qualitative analyses of herbal raw materials are summarized. Spectral correlation coefficient and technique for order preference by similarity to ideal solution (TOPSIS) method were used to evaluate the quality from different producing areas of *Cordyceps* to find the most suitable growing region (Sun et al., 2019). Non-medicinal parts can also be used for origin identification. Different botanical parts of *Gentianae radix et rhizoma* were compared, and researchers found that leaves were the optimal material for geographical characterization (Wang et al., 2018; Liu et al., 2020). The findings illustrate the differences between medicinal and non-medicinal parts at the spectrum level.

The evolution of equipment produces more possibilities in classification. From the articles we searched, the prediction accuracy of IR or Raman spectroscopy techniques reached an acceptable level compared with chromatography methods (Chen et al., 2008; Wang et al., 2020). With unique advantages in heterogeneous sample detection due to the depth-profiling function, FTIR-PAS was first employed in *Cordyceps sinensis*, coupled with PNN (Du et al., 2017). Portable spectrometers are developing. FT-NIR and MicroNIR spectrometer succeeded in identifying four origins of *Salvia miltiorrhiza*. MicroNIR spectrometers had worse performance due to limited spectral information (Sun et al., 2020).

### 4.3 Grade discrimination

The grade of herbs is further subdivided according to their quality discrepancies, which depend on their growing conditions (wild-grown or cultivated, cultivation age, etc.) or parts. The difficulty of grade classification lies in the current form of herbs. The herbal raw materials are processed into powder before they enter the markets. Gad and Bouzabata (2017) failed to discriminate Turmeric powder bought from different commercial stores with various grades using FTIR. The possible reason is FTIR spectra aren’t sensitive to the same species whose phytochemical constituents are the same but in different concentrations.

Parts discrimination against *Ginseng* is meaningful from both academic and commercial points of view. The determination of powdered products is still a problem needed more improvements. DR-NIR spectra were employed to classify different parts of *Ginseng* powder, considering the granularity of the powder. ATR-FTIR spectra were analysed to reveal the difference from molecular functional groups, whose score plots of PCA disclosed a regular and gradual difference in each part (Wu et al., 2011). After suitable normalization methods, ATR-FTIR spectra showed potential in this aspect (Lee et al., 2017). To distinguish wild-grown and cultivated herbs, penalized discriminant methods (Zhu and Tan, 2016) and Adaboost M1 algorithm (Chen et al., 2021) were proposed, both of which had higher computational efficiency and classification accuracy after data pre-processing and variables selection.

### 4.4 Adulteration detection

Low-quality herbs can be pretended to be high-quality when the limited supply cannot meet the increasing demand. Driven by benefit, illegal traders disobey laws to make counterfeits. The adulteration of herbs involves intentionally adding other low-cost or non-pharmaceutical raw materials with a similar appearance to replace or remove certain ingredients without the buyers’ knowledge. The abuse of adulteration leads to severe problems, such as unfair trade competition, public health risks, and social issues. Promising analytic methods are needed to identify the counterfeits, which avoids commercial fraud and guarantees medicine safety.

The application of vibrational spectroscopy techniques in adulteration detection is summarized in **Table 3**. The functional group regions (4000-1300 cm^-1^) have better capability to detect authentic *Astragali radix* (Zhang and Nie, 2010). The inferiority of unsupervised methods as indicated in either MIR or NIR spectroscopy (Liu et al., 2019; Yang et al., 2020) due to poor capability to extract effective information. Data pre-processing, variables selection, IR spectroscopic tri-step identification approach (Chen et al., 2015; Chen et al., 2018), and more supervised algorithms (Shao et al., 2016; Chen et al., 2019; Zhao et al., 2019), which aimed at reducing uncorrelated spectral information, were studied to reach satisfactory results, from adulterated binary samples to adulterated quaternary samples (Nie et al., 2013; Liu et al., 2019). We notice that it is difficult to transfer the models based on small samples directly to other samples because of the limitations of the representativeness of small samples and analytical techniques, as well as the various presence of adulterated chemical components (Li et al., 2020).

A silver nanoparticle wiper as a SERS substrate based on filter paper was made to distinguish nine kinds of dyes adulterated in herbs (Dan et al., 2015). The gold nanorods SERS-based approach functionalized with mono-6-thio-cyclodextrin (HS-β-CD) enhanced the detection capability by strengthening the chemistry interactions (Wu et al., 2018). The fabricated substrate and chemometrics methods (Lin et al., 2018) can improve detection sensitivity. Furthermore, Applying the portable substrate and Raman spectrometer is anticipated to achieve *in situ* detection (Liu et al., 2018).

As we refer to above, adaptive models’ establishment faces challenges in the complex composition of adulteration. In practical application, we need to judge whether the herbal medicine is adulterated and what the impure substance is the next step. Hence, we recommend developing untargeted identification that is advantageous for solving authentication problems (Li et al., 2017; Chen et al., 2020a). Expansion of the samples’ scales and optimization parameters of models are effective means to learn more features from the target class.

### 4.5 Critical quality attribute detection

The influence of the factors mentioned above on the quality of herbs can be basically reflected in critical quality attributes. Critical quality attribute detection, linked to efficacies, has been subjected to more application prospects. The significance of detection is to ensure that the quality of herbs meets the standards for entering the market or has a uniform content consistent in the manufacturing process. We divide critical quality attributes into three categories, active medicinal ingredients, bioactive components, and other regulated indices (moisture, ash, etc.).

The ingredients in herbs, as one of the qualitative evaluation indexes, are listed in the pharmacopeia. Conventional analysis methods require strict extraction and purification. Other regulated indices that illustrate quality, as well as purity, need complicated and laborious operations. In many cases, IR and Raman spectroscopy techniques have been applied successfully, regarded as green and rapid technologies without reagent contamination, which is practical for achieving digital detection.

More than 80% of the studies adopted NIRS, demonstrating the advantages of NIRS in multi-component quantitative detection. PLS is the most popular in multi-component quantitative detection because it can reveal information for the dependent variable as well as reduce the dimensions of the spectral matrix. NIRS-PLS model was applied successfully in the prediction of the total ash and acid-insoluble ash of *Prunellae spica* (Rao and Xiang, 2009), glycyrrhizic acid of *Puerariae lobatae radix* (Mohri et al., 2009), active medicinal ingredients of *Astragali radix* (Zhan et al., 2017), *Paeoniae radix alba* (Luo et al., 2008), *Amomum villosum* (Guo et al., 2021), *Morindae officinalis radix* (Hao et al., 2020), *Dipsaci radix* (Du et al., 2017), and *Notoginseng* (Chen and Sørensen, 2000). These studies also further explain the factors that affect the content of the detection indexes. But sometimes NIRS-PLS caused over-fitting and low precision using full-wavelength spectra (Yang et al., 2003; Yan et al., 2020). The possible reasons could be (1) a limited number of samples. The range of component content distribution is narrow, which results in those differences among various quality are not significant enough to train the robust linear models. (2) low concentration of target components. NIRS proved unsuitable for content lower than 0.1%. Mid-infrared spectroscopy (MIRS) was regarded as a better predictor for analysing low concentration and NIRS utilized the complementarity (Liu et al., 2020).

Due to the fingerprint regions of MIRS and Raman spectroscopy, detection can also be achieved by analysing characteristic peaks. Pei et al. (2008) used the correlation analysis in *Epimedii folium* based on the peak at 1259 ± 1 cm^-1^. The curcumin weight ratio formula was put forward based on band intensity ratios of Raman spectroscopy, analysing different layers of turmeric roots (Peng et al., 2015). The peak intensity at 727 cm^-1^ of berberine was observed in *Coptis chinensis* and *Phellodendron amurence* using SERS (Zhao et al., 2014). TLC-SERS captured the detectable signals, Raman intensity (I_708_/I_728_), which served as the evaluation index in *Coptidis rhizoma* to discriminate and determine berberine and coptisine (Gu et al., 2018).

**Table 4** displays a series of studies regarding quantitative detection that has been carried out by the IR and Raman spectroscopy techniques. The information about techniques, target ingredients, and data processing of concrete herbal plants is available in **Table 4**. Bioactive components, like polysaccharides (Chen et al., 2012; Bu et al., 2013; Ma et al., 2018), flavonoids (Lau et al., 2009; Arslan et al., 2018), alkaloids (Chan et al., 2007; Qi et al., 2018), and antioxidant activity (Wong et al., 2015; Yi et al., 2020), extracted from herbs contribute to sensory quality and efficacy, evaluated by IR and Raman spectroscopy techniques. Pre-treatment, feature selection, non-linear regression methods, and data fusion were tried to improve prediction results. The SVM model presented good generalization performance for *Epimedii folium*, with its R^2^ of more than 0.9 after extracting the feature wavelengths by GA (Yang et al., 2017a). The models built by PLSR and ANN were compared to predict the medicinal ingredients in rhubarb samples (Xue et al., 2018) and *Lonicerae japonicae flos* (Jintao et al., 2021), concluding that models preferred for different components were not the same. There is no doubt that the use of portable spectrometers promotes the application of IR (Wang et al., 2017) and Raman spectroscopy (Wong et al., 2015).

**Table 4:** Summary of quantitative analyses of herbal raw materials based on IR and Raman spectroscopy.

Herbal plants	Technique	Target ingredients	Data processing	Ref.
*Ganoderma*	MIR/NIR	Polysaccharide	VN/BC+ iPLSR/mwPLSR	Ma et al. (2018)
	NIR	Polysaccharides, triterpenoids	MSC/SNV/FD/SD+PLSR/RBF	Chen et al. (2012)
*Astragalus membranaceus*	MIR/NIR	Astragaloside IV, total astragalosides	SG/FD/SD/MSC/SNV+PLSR+data fusion	Liu et al. (2020)
	FT-NIR	Calycosin-7-glucoside, astragaloside	SG/FD/SD/MSC/SNV/ND+PLSR	Zhan et al. (2017)
*Panax ginseng*	NIR	Panaxadiol saponins, panaxatriol saponins, ginseng polysaccharide	MSC/SG/ND+PLSR	Bu et al. (2013)
	FT-NIR	Total main ginsenosides	OSC/FD+PLSR	Huang et al. (2011)
*Panax notoginseng*	NIR	Ginsenosides Rg_1_, R_e_, Rb_1_, R_d_, total ginsenosides	MSC/SNVD+PCA/PLSR	Chen and Sørensen (2000)
	NIR	Panax notoginseng saponins R_1_, ginsenosides Rg_1_, Rb_1_, R_d_, total Panax notoginseng saponins	FD/SD/VN/SLS/MMN/MSC/COE+PLSR	Yang et al. (2003)
*Glycyrrhiza*	NIR	Glycyrrhizin	MSC/SNVD+PCA/PLSR	Chen and Sørensen (2000)
	NIR	Lliquirtin, glycyrrhizic acid	FD+VIP/CARS/MC-UVE/PSO/GA+PLSR	Zhu et al. (2018)
*Pueraria lobata*	NIR	Glycyrrhizic acid	FD/SD+PLSR	Mohri et al. (2009)
	NIR	Puerarin, daidzin, total isoflavonoid	SD/TD/DT/SNV/MSC/SG+PLSR	Lau et al. (2009)
	FT-Raman	Total phenolic content, antioxidant capacities	Norm+PLSR	Wong et al. (2015)
*Epimedium*	NIR	Epimedin A, epimedin B, epimedin C, icariin, moisture contents	FD/SG+GA+PLSR/SVM	Yang et al. (2017a)
	FTIR	Total flavonoids, total content of epimedin A, epimedin B, epimedin C and icariin	Correlation analysis	Pei et al. (2008)
*Paeonia lactiflora*	NIR	Paeoniflorin, albiflorin, benzoylalbiflorin	MSC/SG/FD/SD/TD+MPLS/PLSR/PCR	Luo et al. (2008)
*Poria cocos*	NIR	Polysaccharides, antioxidant activity (DPPH, FRAP, ABTS)	MSC/SNV/Smoothing/FD/SD+ PSO/GA/CARS+PLSR	Yi et al. (2020)
	ATR-FTIR	Poricoic acid A, dehydrotrametenolic acid, dehydropachymic acid, pachymic acid, dehydrotrametenolic acid	SG+PLSR	Wang et al. (2020)
*Lonicera japonica*	NIR	Chlorogenic acid, isochlorogenic acid A, isochlorogenic acid C	FD/SD/MSC/SLS/MMN/VN/COE/SG+ PLSR/ANN	Jintao et al. (2021)
*Salvia miltiorrhiza*	NIR	Tanshinone II A, cryptotanshinone, tanshinone I, salvianolic acid B, antioxidant activity	SNV/MSC/SG/FD+ iPLS/ Bi-PLS/CARS+PLSR	Sun et al. (2020)
*Prunella vulgaris*	NIR	Total ash, acid-insoluble ash	COE/SLS/VN/MMN/MSC/FD/SD+PLSR	Rao and Xiang (2009)
*Coptis*	FT-IR/NIR	Eight alkaloids	Smoothing/MSC/SNV/FD/SD+PLSR+ data fusion	Qi et al. (2018)
	TLC-SERS	Four protoberberine alkaloids	Peaks analysis	Gu et al. (2018)
*Phellodendron chinense*	NIR	Berberine, total alkaloid	MSC/SNV/SG+PLSR	Chan et al. (2007)
*Gentiana*	FTIR	Gentiopicroside, total of four iridoids	FD/SD/SNV/MSC+PLSR	Qi et al. (2016)
*Verbena officinalis*	NIR	Verbenalin, verbascoside	FD/SD+PLSR	Pezzei et al. (2017)
*Typha*	NIR	Typhaneoside, isorhamnetin-3-O-glucoside	MSC/SNV/WDS/SG+CARS+PLSR	Sun et al. (2019)
*Rheum*	NIR	Chrysophanol, aloe-emodin, rhein, emodin, physcion	SG/VN/MMN/MSC/SLS/COE/FD/SD+ PLSR/ANN	Xue et al. (2018)
*Chrysanthemum morfolium*	NIR	Absolute contents of six Q-markers with anti-inflammation activity	SNV/MSC/DT/oneDC/Smoothing+ PLSR/RF/nu-SVR/BPANN	Ding et al. (2016)
*Lycium barbarum*	FT-NIR	Total flavonoid content, total anthocyanin content, total carotenoid content, total sugar, and total acid	SNV/MSC+Si-PLS/Bi-PLS/GA+PLSR	Arslan et al. (2018)
*Andrographis paniculata*	NIR	Andrographolide, deoxyandrographolide, dehydroandrographolide, neoandrographolide, moisture, ash content, and alcohol-soluble extract	SNV/MSC/FD/SD/SG+PLSR	Lai et al. (2018)
*Codonopsis*	NIR	Polysaccharide	SNV/MSC/FD/SG+CARS/SPA/iPLS+PLSR	Wang et al. (2017)
*Dipsacus asper*	FT-NIR	Loganic acid, chlorogenic acid, caffeic acid, loganin, isochlorogenic acid B, isochlorogenic acid A, isochlorogenic acid C, asperosaponin VI	SNV/MSC/FD/SG+PLSR	Du et al. (2017)
*Amomum villosum*	FT-NIR	Camphor, borneol and bornyl acetate	SNV/MSC/FD/SD/SG+PLSR	Guo et al. (2021)
*Prunus mume*	FT-NIR	Neochlorogenic acid, chlorogenic acid, rutin, hyperoside and isoquercitrin, quercitrin, quercetin and kaempferol	MSC/SNV/FD/SD/SG+PLSR	Yan et al. (2020)
*Morinda officinalis*	FT-NIR	Fructose, glucose, sucrose, fructooligosaccharides and iridoid glycosides	MSC/SNV/FD/SD/SG+PLSR	Hao et al. (2020)
*Paeonia lactiflora*	DR-NIR	Moisture content, albiflorin, paeoniflorin	MSC/SNV/Norm+HCA-RC/SSC+PLSR	Ma et al. (2020)
*Zingiber officinale*	FT-NIR	Zingerone, 6-gingerol, 8-gingerol, 6-shogaol, 10-gingerol	SNV/MSC/FD/SD/SG+PLSR/GA-CP-ANN	Yan et al. (2021)
*Curcuma*	Raman	Curcuminoids	BC+linear fitting	Peng et al. (2015)
*Coptis* and *Phellodendron*	SERS	Berberine	Peaks analysis and linear fitting	Zhao et al. (2014)

In short, different strategies should be selected according to purposes and requirements in production practice (Yan et al., 2021). Spectroscopic techniques have the outstanding advantage of simultaneous multi-component analysis. The rapid determination of herbal raw materials using IR and Raman spectroscopy techniques has high practical value in the pharmaceutical industry. Meantime, these techniques provide reliable technical support for the evaluation of the critical quality attributes and on-line measurements during the production process.

## 5 Processing quality control

**Figure 4:**
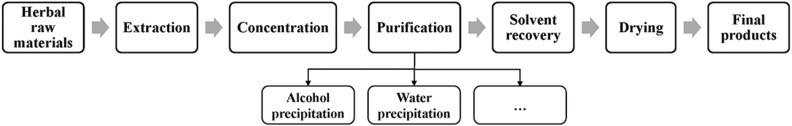
Typical production processes of herbs.

Processing is an indispensable procedure before herbs enter the market, aiming at enhancing efficacy and reducing side effects. The internal compounds of herbs are usually diverse and ambiguous. The process analytical technology (PAT) guidance was issued by the American Food and Drug Administration in 2004 for processing quality control. PAT uses a series of tools and means to realize real-time analysis and feedback control during industrial production to ensure a controllable production process and optimal product quality. **Figure 4** shows the typical production processes of herbs. The whole process involves multiple unit operations. There are four methods to monitor critical process parameters (CPPs): off-line, at-line, on-line, and in-line (Cortés et al., 2019).

Traditionally, the endpoint of these processes relies extensively on empirical experience, conventional off-line analysis methods, or fixing the process parameters like temperature, time, and solvent concentration. Moreover, herbs as natural products have batch-to-batch variability. It cannot ensure product quality and batch-to-batch consistency with identical process settings. Vibrational spectroscopy techniques can realize on-line, real-time, and rapid detection of the internal quality of herbs during the processing, making the operation more controllable and understanding.

To comprehensively understand and optimize the procedures, 2D-IR is applied to explore the chemical mechanism by analysing the characteristic peaks of compounds (Chen et al., 2007; Guo et al., 2016; Adib and Abdullah, 2018). For example, the temperature-perturbation 2D-IR spectra can be applied to determine and optimize parameters during thermal processing, knowing the change rules of compounds in different stages (Chen et al., 2016; Wu et al., 2019; Yang et al., 2020).

NIRS has become a hot research topic in the field of process analysis because of its characteristics of fast, non-destructive, and pollution-free analysis. The NIR light has good transmission characteristics in optical fiber, through which the collected signals can be transmitted to spectrometers far away from the production site in real-time. Experiments were conducted on a laboratory scale, verifying the NIR for detecting CPPs. The extraction (Wang et al., 2015; Hu et al., 2017; Li et al., 2018; Lyu et al., 2018; Zhong et al., 2018; Hua et al., 2021) and purification processes (Luo et al., 2017; Huang and Qu, 2018) based on the NIRS-PLS model, which showed the application potential of NIR in PAT analysis. The relationship between NIRS and CPPs content was more complicated, and non-linear prediction models, such as SVM, ANN, and CNN, may be more suitable (Qu et al., 2004; Liu et al., 2017). Usually, the strong absorption peak of water is usually removed to eliminate the negative impact. Gao et al. (2021) established a reconstructed spectrum based on PCA to monitor the *salvianolic acid B* in the water precipitation process of *Salvia miltziorrhiza bge*. They regarded water as a probe to understand better and visualize the extraction process. The unreliability graph methodology was innovatively proposed as a release strategy in *tanshinone* extract powders after the establishment of NIRS-PLS model (Shi et al., 2019).

So far, most PAT research has been conducted on lab-scale equipment where some experimental conditions are easy to control. Due to the complexity of the actual production processes, further research is needed to transfer models from the laboratory to the factory and to set up experimental field facilities. Recently, some scholars collected samples from the production line, which is more consistent with the actual production (Zhao et al., 2020). Two hundred samples were collected in the product line of *Tanreqing* injection to determine the CPPs. Gaussian process model achieved better performance than PLS and LS-SVM and showed the best interpretability (Li et al., 2019). Yang et al. (2017b) designed an external loop to make extracts of *Flos Lonicerae Japonicae* flow into NIRS on-line measurements. A new algorithm of synergy interval PLS with genetic algorithm (Si-GA-PLS) was proposed for modeling, the R^2^ of 0.9561 for total acid reached. The NIR sensors installed in the production line automatically and continuously measured the CPPs (Huang and Qu, 2011; Zhang et al., 2019). The five main saponins in the elution process for purity were monitored using CNN based on in-line NIR. CNN model obtained better results than PLS models with the ‘automatic pre-processing’ functions of the convolutional layer (Yan et al., 2020). As for Raman spectroscopy technique, Jin et al. (2020) first trained the RS-CARS-PLS model to monitor the simulated extraction process for *Wenxin* granule manufacture. However, this method had a relatively high LOD, which could not detect saccharides with low concentration.

PAT improves the understanding of the production process and products and the control during the production process, ensuring the quality of products. On-line or in-line monitoring with vibrational spectroscopy techniques is more practical for the quality control of the process. NIRS technique is considered a promising method in PAT analysis because it significantly saves workforce and time, owing to its good multi-component prediction performance and fiber transmission characteristics. NIRS-PLS model meets the basic requirements in assays. Non-linear models can achieve higher accuracy while increasing the computational cost. Therefore, PLSR is more practical when we care more about detecting speed than high accuracy.

## 6 Quality and safety inspection of patent herbal products

Patent herbal products (PHPs) refer to patent herbal medicines for treatment and herbal nutraceuticals for health care. Herbs are usually used as decoctions by boiling them with water. Decoctions are easier to be absorbed while not convenient to carry and store. The PHPs are developed instead of decoctions. PHPs with easy-to-use characteristics contain pills, granules, and preparations used as clinical medicines or dietary supplements, which breaks through the traditional treatment way of herbs and expands the application scope. PHPs are made up of multiple herbal extracts and excipients. The quality and safety of PHPs constitute a significant concern to ensure their efficacy.

### 6.1 Index components detection

Knowing the index components of PHPs can evaluate the quality and provide a reference for dosage. Spectroscopic techniques are applied as rapid, non-invasive methods, requiring minimal sample pre-treatment. The successful distinguishing of thirty-six commercial brands of *Ganoderma lucidum* (Sun et al., 2001) and consistent characteristic peaks in PHPs compared with individual herbs (Bansal and Reddy, 2018) based on FTIR illustrated the evaluation can be done. Chen et al. (2016) adopted FTIR microspectroscopic imaging to collect pixel spectra. The direct and simultaneous recognition of multiple organic and inorganic ingredients in PHPs was achieved by comparing the reference spectrum and calibration set.

NIRS-PLS model was employed to determine two different sample presentations originating from a turmeric capsule and powder, obtaining ideal results in powder samples (Kasemsumran et al., 2014). The concentration of *Coptis chinensis* in suppositories was also predicted (Teraoka et al., 2012). Three presentations, capsule shells, contents, and intact capsules, of *Yaobitong* capsule, were analysed using NIRS-LSSVM model (Si et al., 2021). MIRS-PLS model was used to predict feeding levels of PHPs by detecting the excipient content (Guo et al., 2016) as well as the antioxidant activity of mixed herbal infusions (Venetsanou et al., 2017). Laser-induced breakdown spectroscopy (LIBS) with element information and MIRS with molecular information was fused to classify the compound *Salvia miltiorrhiza* by RF discrimination models (Liang et al., 2020).

Because the predictive performance of the model will be affected by samples (formula, batch, manufacturer, etc.) and algorithms (pre-treatment, feature extraction, modeling), the scope of the calibration set should cover the test set. Therefore, the robustness of models requires sufficient representative samples, still long-term research work.

### 6.2 Counterfeiting and adulteration detection

PHPs have milder effects than western medicine in a slow curative effect with fewer side effects. Demand is increasing due to the growing popularity of herbal dietary supplements and clinical medicines. Synthetic drugs and regulated or toxic substances are added undeclared to PHPs for illegal profits, which results in consumers being vulnerable to counterfeiting and adulteration.

NIRS coupled with PLS-DA distinguished PHPs adulterated with sibutramine with a correct classification of 100%. Four variables selected by MLR-SPA were executed to build a quantitative model (Da Silva et al., 2015). Feng et al. (2014) improved the reverse correlation coefficient (RCCM) for threshold settings to test antidiabetic PHM illegally added with synthetic drugs. MIRS presents a ‘fingerprint’ with high sensitivity and selectivity in terms of sample peaks and peak intensities. MIRS showed the best performance among MIRS, NIRS, and Raman spectroscopy techniques in detecting PHM adulterated with sibutramine and phenolphthalein (Rooney et al., 2015). The performance of fused data using MIRS and NIRS was poorer than MIRS data alone (Deconinck et al., 2017). NIRS data failed to add valuable information according to the loading analysis.

Observing and comparing characteristic peaks in MIRS and Raman spectroscopy is also effective means of identification due to the significant differences between PHPs and adulterated products (Mateescu et al., 2017). Slimming herbal products, adulterated with illegal additives, were discriminated against by constructing synchronous and asynchronous maps (Miao et al., 2017). Univariate calibration (De La Asunción-Nadal et al., 2017) and mathematically fortified spectra (Walkowiak et al., 2018) based on ATR-FTIR offer a fast, eco-friendly, and cheap alternative for adulterations identification with good analytical features. The local straight-line screening (LSLS) algorithm, newly proposed in 2007 and modified in 2009, has proved the feasibility of detecting the illegal incorporation of synthetic drugs in PHPs after careful observation of the shape of the spectral line (Lu et al., 2007; Zhu et al., 2009). TLC-SERS was established and used to detect adulterated PHPs for curing diabetes (Zhu et al., 2014), cholesterol (Zhu et al., 2017), and sexual performance (Dao et al., 2019).

Current microscopic and chemical identification above showed the feasibility and application prospect of IR and Raman spectroscopy techniques in the quality and safety of PHPs. Various PHPs can be detected without complex procedure extraction of marker compounds. Vibrational spectroscopy techniques can be applied as a preliminary evaluation of suspicious PHPs, even though their current LODs are inferior to traditional chromatographic methods. The identified PHPs are confirmed using specific chromatographic methods afterward. Spectroscopy analysis methods are expected to be widely applied if the entire experimental procedure can be optimized, standardized, and automated. The development trend of small-type and portable spectrometers makes mobile laboratories feasible, conducted in the open market and throughout the herb distribution channel.

## 7 Challenges and future remarks

The above review summarized the application of IR and Raman spectroscopy in the quality and safety inspection of herbs across the whole process. Spectral differences are captured and enlarged by various data processing. Evaluating and controlling the quality of herbs based on spectral techniques can save workforce and time, and effectively evaluate the efficacy of herbs. Vibrational spectroscopy techniques combined with chemometrics provide new monitoring concepts with data acquisition and processing automatic, which promote the digital detection of herbs.

The challenges we face are establishing more steady and robust models and achieving online monitoring in real production practice. Research trends focus on signal enhancement and effective information extraction for ideal prediction accuracy. In the future, the application of IR and Raman spectroscopy techniques in herbs has the potential to drive the development of the industry, which contributes to digital detection for quality and safety inspection of herbs across the whole process. **Table 5** summarizes the challenges and future remarks.

- IR and Raman spectroscopy techniques are complementary, so the combined techniques can characterize the sample with a more comprehensive description. Multi-source spectral techniques or spectral coupling with chromatography are both investigated. Images are also used to supplement spectral information, which presents distribution characteristics of herbal components and improves the performance of models in identification or detection. Image information can be obtained by converting spectral matrixes into an image matrix or selecting imaging equipment, such as microscopic infrared imaging and confocal Raman imaging systems. We could see that multiple hyphenated techniques facilitate data fusion, which has been applied to detect the chemically active components in herbs and characterize them more comprehensively and systematically.
- The widespread pattern recognition or quantitative prediction models are mainly based on machine learning, the more traditional but classic models like PCA, LDA, PLSR, and SVM. With the boost of artificial intelligence, DL algorithms for data processing to enlarge the amount of data would be considered, excavating deeper into the validity of spectral data. DL with self-learning and migration has advantages in feature mining of herbs, which makes it feasible to characterize herbs with similar characteristics (same medicinal parts, same genera, etc.). Indeed, maintenance of the model, including updates and expansions, is also indispensable.
- So far, the achievements were obtained chiefly in the laboratory with specific operating conditions and application restrictions. To realize the final aims that use them in the actual production environment, the improvement of sample preparation methods and the development of portable instruments need to be concentrated on and gradually progress, giving full play to the advantages of spectral technology. As a fingerprint technique, building spectra databases of herbs can broaden their application scenarios. The spectroscopic database can be gradually completed through the continuous accumulation of experiments. Breaking the independence of spectral detection of herbs further quickly and effectively improves herbs identification accuracy.

**Table 5:** Brief summary of challenges and future remarks of herbs.

Challenge	Current situation	Solution
Limited number of samples	Data from single spectrometer or form	Multi-source spectral data fusion
		Images and spectra data fusion
		The use of non-medicinal parts
Poor performance in model stability and transfer	Simple line fitting & traditional but classic models based on ML	Mining for effective information among big data
		Make use of deep learning for self-learning and migration
		Good model management: expand and update
Less application in actual production	Carried out in the laboratory scales in defined conditions	Develop stable portable instruments
		Analyze samples in the production line for applicability
		Build spectra databases for better interpretation

## 8 Conclusion

The efficacies of herbs have been expanded from medicine to food, health care products, daily necessities, and other fields. The quality and safety of herbs have been widely concerned. The application of IR and Raman spectroscopy in the quality and safety inspection of herbs has feasibility, which could be employed across the whole process of herbs with excellent application prospects. IR and Raman spectroscopy techniques are sufficient to meet the requirements due to their advantages of fast speed, micro- or non-damage, no environmental pollution, and outstanding ability of online detection.

Detection tasks may benefit from increased model sensitivity. How a model is integrated into the practical workflow is another crucial consideration for promoting digital detection, as algorithms can be deployed in various ways. It is a promising way to integrate the trained models into software to guide decision-making. With the development of spectroscopic instruments and advanced algorithms, the accuracy and efficiency of the IR and Raman spectroscopy techniques have been improved little by little. Vibrational spectroscopy techniques have shown great application advantages, which serve as solid support for promoting digital detection, then building intelligence and automation of herb products factories, boosting the digital transformation of the herb industry.

## Author contributions

RC: Investigation, Writing-Original draft, Visualization; FL: Investigation, Writing-Original draft, Writing-Review & editing; CZ: Writing-Original draft; WW: Writing-Review & editing; RY: Writing-Review & editing; YZ: Writing-Review & editing; JP: Writing-Review & editing; WK: Conceptualization, Writing-Review & editing; JH: Conceptualization, Writing-Review & editing, Supervision. All authors contributed to the article and approved the submitted version.
